# Iron Homeostasis During Pregnancy: Maternal, Placental, and Fetal Regulatory Mechanisms

**DOI:** 10.1146/annurev-nutr-061021-030404

**Published:** 2023-05-30

**Authors:** Veena Sangkhae, Allison L. Fisher, Tomas Ganz, Elizabeta Nemeth

**Affiliations:** 1 Center for Iron Disorders, David Geffen School of Medicine, University of California, Los Angeles, Los Angeles, California, USA; 2 Endocrine Unit and Nephrology Division, Massachusetts General Hospital, Harvard Medical School, Boston, Massachusetts, USA

**Keywords:** pregnancy, iron, hepcidin, placenta, iron deficiency, inflammation

## Abstract

Pregnancy entails a large negative balance of iron, an essential micronutrient. During pregnancy, iron requirements increase substantially to support both maternal red blood cell expansion and the development of the placenta and fetus. As insufficient iron has long been linked to adverse pregnancy outcomes, universal iron supplementation is common practice before and during pregnancy. However, in high-resource countries with iron fortification of staple foods and increased red meat consumption, the effects of too much iron supplementation during pregnancy have become a concern because iron excess has also been linked to adverse pregnancy outcomes. In this review, we address physiologic iron homeostasis of the mother, placenta, and fetus and discuss perturbations in iron homeostasis that result in pathological pregnancy. As many mechanistic regulatory systems have been deduced from animal models, we also discuss the principles learned from these models and how these may apply to human pregnancy.

## IRON HOMEOSTASIS DURING PREGNANCY

### Iron Requirements During Pregnancy

Iron requirements in women of reproductive age are higher than those for men because of the loss of iron in menstrual blood (58). During pregnancy, maternal iron demands further increase from approximately 1 mg/day in nonpregnant females to nearly 7 mg/day in the third trimester of pregnancy. Iron is essential to support development of the placenta and fetus and for pregnancy-related expansion of maternal red blood cell (RBC) mass. In total, over the course of pregnancy, approximately 1 g of additional iron is required to sustain healthy pregnancy. Inability to meet these requirements can result in maternal iron deficiency and iron deficiency anemia, which have been linked to adverse pregnancy outcomes (3). Table 1 summarizes iron balances in menstruating females and during each trimester of pregnancy, with estimates based on a 120-lb (54-kg) woman. Aside from menstrual blood loss, basal iron losses are similar between the nonpregnant and pregnant states and are estimated at 0.8 mg/day (58) or 224 mg over 9 months of gestation. During the first trimester of pregnancy, maternal iron requirements marginally decrease from ~1.3 mg/day to ~0.8 mg/day due to cessation of menstruation (58).

The placenta weighs 223 g on average by the end of the second trimester (26 weeks) (61) and 640 g by term (40 weeks) (8, 60, 139). Assuming placental iron concentration is similar through gestation [71 μg iron/g placenta (8)], approximately 16 mg and 30 mg of iron is needed to support placental growth and development in the second and third trimester, respectively, with the total amount of placental iron at term estimated at ~46 mg (8), although the estimates go as high as 150 mg (68).

Mean fetal weight by 26 weeks is 890 g (2), and on the basis of fetal iron measurements from Widdowson & Spray in 1951 (139), ~60 mg of iron is needed for fetal development in the second trimester. In the third trimester, fetal growth outpaces placental growth and requires an additional 210 mg of iron (2, 139), with the total amount of iron in the fetus at term estimated at ~270 mg.

Maternal RBC mass expansion requires ~450 mg of iron over gestation (13), and assuming a linear increase over the second and third trimesters (69), ~112 mg and ~338 mg of iron, respectively, are required for this adaptation. In addition to the 1 g of iron required for pregnancy, more iron is lost through bleeding during delivery (150 mg on average), but maternal RBC contraction after delivery returns approximately 450 mg to the mother (13), so that the net loss to the mother over gestation is approximately 700 mg of iron (13, 37).

To meet these additional iron requirements during pregnancy, iron absorption from the diet increases and, when available, is mobilized from maternal stores. As iron deficiency is the most common micronutrient deficiency in the world (142), many women of reproductive age are already iron deficient prior to pregnancy, and those from socially disadvantaged populations are disproportionally affected (22). Depending on the cutoffs used to define iron deficiency, up to 40% of women in the first trimester of pregnancy in the United States may be iron deficient (11). This has led the American College of Obstetrics and Gynecologists and the Centers for Disease Control and Prevention to recommend universal oral iron supplementation for pregnant women (11). However, it is important to consider that in medically and nutritionally well-resourced populations, most women are iron replete with sufficient iron stores when entering pregnancy, thus prompting considerations of potential risks of indiscriminate iron supplementation (130).

### Sources of Iron

**Figure 1:**
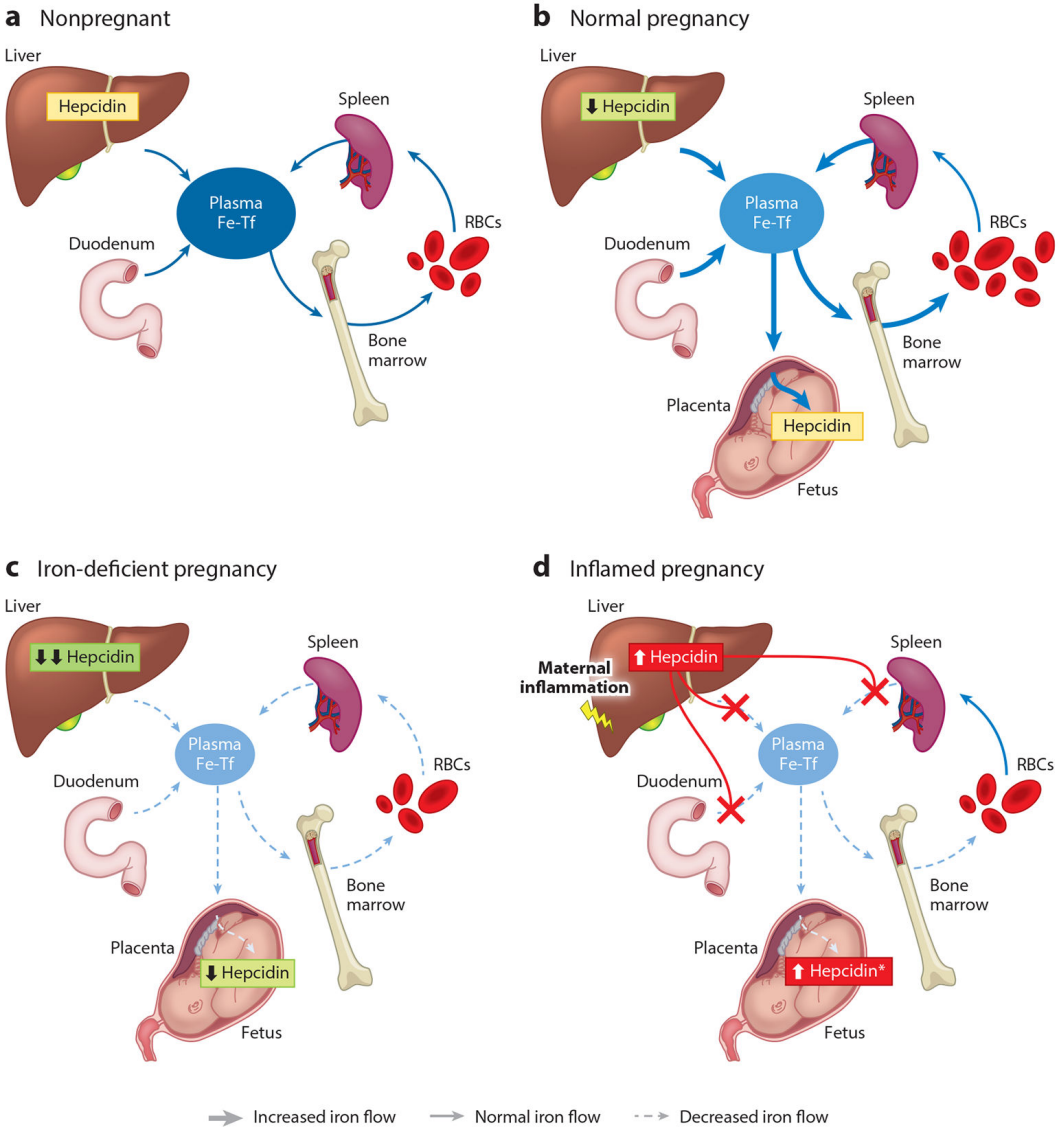
Iron flows in (*a*) normal nonpregnant females, (*b*) normal pregnancy, (*c*) iron-deficient pregnancy, and (*d*) inflamed pregnancy. Iron flows are shown in shades of blue and different arrow sizes, where darker thick arrows indicate increased iron flows and lighter dashed arrows indicate decreased or absent iron flows compared with normal flow. Hepcidin effects are shown in red. The asterisk indicates intra-amniotic infection. Abbreviations: Fe, iron; RBC, red blood cell; Tf, transferrin.

During pregnancy, dietary iron absorption increases significantly (Figure 1). Iron is present in food as (*a*) heme iron, found primarily in animal products in myoglobin and hemoglobin, or (*b*) nonheme iron, typically from plant-derived products. Both heme and nonheme iron are absorbed in the duodenum (proximal part of the small intestine) and share a common pathway out of the enterocyte to extracellular fluid and blood through the iron exporter ferroportin (*SLC40A1*) (54). In this section, intestinal absorption of heme and nonheme iron are briefly discussed in the context of pregnancy. A more extensive review of intestinal iron absorption can be found in Reference 54.

#### Absorption of heme iron.

The proportion of iron in the diet contained in heme is estimated to be ~10%. Since these estimates were developed in the 1980s, the global consumption of meat has dramatically increased (50), suggesting that heme-based iron now represents a larger fraction of iron in the diet. Moreover, heme iron is more bioavailable than nonheme iron (67), making it an important determinant of iron sufficiency in most populations. Using iron isotopes, Young et al. (147) confirmed that heme-derived iron utilization is greater than nonheme iron utilization in nonpregnant and pregnant females. However, the percentage of heme-derived iron utilization was similar between nonpregnant and pregnant females. Furthermore, heme iron utilization was not related to maternal iron status (147). Overall, these data suggest that iron uptake from heme-iron sources is not altered in response to pregnancy.

Despite the importance of heme as a source of dietary iron, the mechanism of heme absorption is not well understood. Heme is thought to be absorbed by intestinal epithelial cells in the duodenum via receptor-mediated endocytosis, then catabolized in the cytoplasm possibly by heme oxygenase to release iron (102), which is then exported on the basolateral surface of enterocytes through ferroportin into the plasma. A significant barrier to our understanding of heme iron absorption is that the intestinal heme transporter has not yet been identified. Studies in rodent models may not be informative about this mechanism because rodents do not absorb dietary heme efficiently (35).

#### Absorption of nonheme iron.

Intestinal absorption of iron from nonheme sources has been extensively characterized. Briefly, luminal ferric iron (Fe^3+^) is reduced by a mucosal ferrireductase (DCYTB) to ferrous iron (Fe^2+^), whereupon it is taken up into the enterocyte by proton-coupled divalent metal transporter 1 (DMT1) with the inward proton gradient generated by cellular proton exporters NHE3 (Na^+^/H^+^ exchanger) and, to a lesser extent, NHE2 (122). In the enterocyte cytoplasm, iron is either stored as ferritin or exported to the plasma through ferroportin. Absorption and utilization of dietary nonheme iron increases during pregnancy (147). Expression of duodenal iron transporters is increased during pregnancy, and both nonheme iron uptake into the enterocyte and export of iron from the enterocyte to the serum are increased (91, 95), at least in part because of decreased systemic hepcidin levels (discussed later).

Supplemental iron, provided in nonprescription and prescription prenatal products, is routinely advised during pregnancy and is even commonly recommended for several months prior to becoming pregnant. Iron in prenatal supplements is typically in the form of iron salts (nonheme iron) providing 26–34 mg of elemental iron per dose (range: 4.5–106 mg/dose) (109). In the United States, more than 75% of pregnant women use prenatal supplements, with use increasing as pregnancy progresses (16). However, as discussed previously, most women in developed countries including the United States are already iron replete before pregnancy, raising questions about whether iron supplementation is necessary in this population. Iron supplementation and the effects of maternal iron status on iron homeostasis and fetal health are discussed later in the review.

### Regulation of Maternal Iron Bioavailability

The hepatic hormone hepcidin regulates the absorption of dietary iron, concentrations of iron in the plasma, and the distribution of iron among organs and tissues (reviewed in 45) (Figure 1). During pregnancy, maternal hepcidin concentrations control the delivery of iron to the placenta and the fetus. In the nonpregnant state, hepcidin is predominantly regulated by iron status, inflammation, and erythropoietic drive (reviewed in 113). However, regulation of hepcidin during pregnancy is not as well defined. As pregnancy progresses, hepcidin levels progressively decrease (Figure 1b). This decrease occurs in both human (36, 40, 134) and rodent (95, 112) pregnancy. In humans, hepcidin levels increase slightly in the first trimester compared with those of nonpregnant women due to cessation of menstruation (58), then hepcidin levels progressively decline in the second and third trimesters (134). Decreased hepcidin allows for increased iron absorption (113) and release of iron from liver stores to maintain circulating iron levels (112) for uptake by the placenta and transfer to the developing fetus. Despite the importance of maternal hepcidin for maternal and fetal health (111), the mechanism of maternal hepcidin suppression remains unknown. On the basis of studies in mouse models, absence of maternal hepcidin results in viable but iron-overloaded offspring; however, elevation of maternal hepcidin concentrations through administration of exogenous hepcidin (111) results in fetal iron deficiency anemia and even death.

#### Hepcidin is regulated by maternal iron status.

Growing evidence suggests that hepcidin regulation by iron status is preserved in pregnancy. Maternal hepcidin positively correlates with maternal serum iron parameters (36) or ferritin (36, 57, 121) and is increased by iron supplementation in humans (40) and rodents (44). In animal models, despite lower hepcidin during pregnancy, iron deficiency further decreased maternal hepcidin (26, 44, 112). Similarly, in a case-controlled human study by Zaman et al. (149), serum hepcidin levels were significantly lower in pregnant women with iron-deficiency anemia compared with those of pregnant women with non-iron-deficiency anemia and healthy pregnant women (Figure 1c).

#### Inflammation and hepcidin during pregnancy.

Inflammation is a potent inducer of hepcidin (98, 99). Although this is well described in the nonpregnant state, the effects of inflammation on iron homeostasis during pregnancy have only recently been examined. Human studies in most healthy pregnancies report no correlation between hepcidin and C-reactive protein or interleukin 6 (121, 124, 134), but the association exists in pregnancies complicated by inflammation and infections (53) (Figure 1d). As in nonpregnant states, iron deficiency and inflammation frequently coexist, vary in relative severity, and will exert opposing effects on hepcidin levels. In animal models, where the effect of inflammation can be isolated, systemic maternal inflammation substantially induces maternal hepcidin expression (39, 112). As mentioned previously, elevated maternal hepcidin even in the absence of inflammation results in adverse pregnancy outcomes (32, 100, 111). Intra-amniotic inflammation may not necessarily increase maternal hepcidin, as was seen with endotoxin administration in macaques where only fetal hepcidin increased (39). Therefore, during pregnancy, inflammation can regulate hepcidin expression; however, the severity and location of inflammation will determine if and whether maternal or fetal iron homeostasis is affected.

#### Hepcidin regulation by erythropoietin/erythroferrone.

Erythropoietin (EPO), a renal and hepatic hormone induced by hypoxic stimuli including anemia, is essential for erythropoiesis and stimulates erythropoietic activity. During pregnancy, EPO synthesis gradually increases (10) because of dilutional anemia, but the effect is blunted by increased blood flow and oxygen delivery to the kidneys, the organs that sense hypoxia and produce EPO (10). Increased EPO levels support the expansion of RBC mass associated with pregnancy. More recently, EPO has been demonstrated to upregulate erythroferrone (ERFE), the erythroid regulator of iron homeostasis (72). ERFE regulates iron homeostasis by sequestering hepcidin inducer bone morphogenetic protein 6 (BMP6), thus lowering hepcidin levels. In nonanemic human and rodent pregnancies, serum ERFE levels are not significantly affected by pregnancy status (138). However, in mouse models of iron-restricted or iron-deficient pregnancy with ensuing anemia, maternal ERFE levels are significantly elevated (111, 115). Elevated maternal ERFE has also been reported in anemic human pregnancies (30). Mechanistic studies in mouse models showed that ERFE knockout mice are fertile (72) and that maternal hepcidin is appropriately suppressed, indicating that ERFE is not the physiological pregnancy hepcidin suppressor. In iron-deficient dams, ERFE deficiency had only very minor effects on maternal erythropoiesis (115). Thus, maternal ERFE may be a useful marker of iron deficiency anemia in pregnancy but does not strongly regulate maternal iron homeostasis.

#### Pregnancy-specific hepcidin regulator.

Maternal hepcidin is profoundly decreased during pregnancy, but many studies measuring hepcidin include iron-deficient pregnancies, confounding our understanding of the effect of pregnancy on hepcidin. In studies including only iron-sufficient pregnancies, as determined by hemoglobin and ferritin, hepcidin was still decreased in the third trimester (62, 124), suggesting the existence of pregnancy-related hepcidin regulation. This concept is particularly supported by a downward shift in the correlation curves of serum hepcidin versus serum ferritin from the first to second trimester in human pregnancy (57). In both trimesters, serum hepcidin positively correlated with serum ferritin, but hepcidin levels were nearly 10-fold lower in the second trimester over a similar range of ferritins. The timing of maternal hepcidin suppression also suggests pregnancy-specific regulation of hepcidin. In both humans and rodents, hepcidin levels decrease prior to a decrease in maternal iron stores (7, 112). The identity of the pregnancy-specific hepcidin regulator(s) remains to be established.

#### Hormonal regulation of hepcidin.

Estrogen, progesterone, and prolactin are hormones that increase substantially over the course of pregnancy (118); all have been implicated in hepcidin regulation and thus could modulate iron homeostasis during pregnancy.

#### Estrogen (17β-estradiol).

Estrogens increase approximately 10-fold over the course of pregnancy, with levels peaking in the third trimester in humans at approximately 70 nM (118). A number of studies have described a role for estrogen in iron metabolism. An early study in 2009 was performed in largemouth bass, where experimental exposure of fish to 17β-estradiol (E2) reduced the constitutive expression of hepcidin-1 in the liver (106). Since then, several mouse and human studies have supported a role of estrogen in hepcidin regulation but report conflicting results on the direction of hepcidin change. A functional estrogen response element has been reported in the hepcidin promotor region (66, 145).

In some studies, E2 was a negative regulator of hepcidin. Hepcidin was suppressed in vitro in human hepatic cell lines Huh7 and HepG2 treated with 100 nM of E2, and the effect was blocked by an E2 receptor antagonist ICI182780. Hepcidin was also suppressed in vivo in mice following E2 injection (145). In ovariectomized mice, where estrogen is low, hepcidin levels were elevated compared with those of sham surgery mice (66). In female rats fed a low-iron diet, multiple treatments with E2 to mimic pregnancy conditions resulted in a dose-dependent increase in liver iron concentrations, and although hepcidin was not measured in this study, outcomes were consistent with decreased hepcidin levels (65). In humans, stimulation of endogenous estrogen production in nonpregnant women resulted in an approximately threefold decrease in hepcidin levels (80). However, other studies report that estrogen does not affect or may even induce hepcidin expression. Treatment of human hepatoma cells with increasing E2 doses resulted in no change in hepcidin (150) or in increased hepcidin expression (70). Ovariectomized mice had decreased hepcidin levels along with increased ferroportin expression and increased serum and liver iron concentrations (70), suggesting that estrogen (or other hormones produced by the ovary) functions as a hepcidin inducer.

Although these studies were performed in highly simplified models in vitro, or in a nonpregnant state in vivo, their findings are relevant to pregnancy, as estrogen is greatly induced during pregnancy. As estrogen is essential for healthy pregnancy and low third-trimester urinary estriol is associated with fetal death or anencephaly (94), studying the specific role of estrogen in iron metabolism during pregnancy is challenging but important because of possible interactions of estrogen with other pregnancy hormones. In summary, the data on the role of estrogen in hepcidin regulation and iron homeostasis are inconclusive, highlighting the need for more definitive studies.

#### Progesterone.

Progesterone increases approximately fivefold over the course of pregnancy (118). However, progesterone treatment in zebrafish (5 μM) increased hepcidin mRNA expression 10-fold, and in the human hepatic cell line HepG2 (30 μM) it was increased 30-fold. This increase in hepcidin expression is reported to occur not through the classical nuclear progesterone receptor but through the membrane-bound progesterone receptor PGRMC1 (progesterone receptor membrane component 1) (84). During pregnancy, however, overall hepcidin levels decrease. Among the possible limitations of these studies of progesterone effects are the short timing of the experiment (8 h) and the high concentrations of progesterone used. Progesterone in the third trimester of pregnancy is approximately 400 nM.

#### Prolactin.

During healthy pregnancy, serum prolactin levels steadily increase throughout gestation starting in the first trimester (105). Treatment of HepG2 cell lines with prolactin reduced hepcidin mRNA levels (137), and treatment of hyperprolactinemic women with the prolactin-reducing drug bromocriptine mesylate increased hepcidin levels (137), whereas another study found that pathological hyperprolactinemia did not influence serum hepcidin-25 levels (80). Both studies were performed in the nonpregnant state. Additional studies are required to determine if there is a definitive role for prolactin in regulating hepcidin in pregnancy.

#### Other possible regulators of hepcidin during pregnancy.

Several other factors may contribute to hepcidin regulation during pregnancy, but the extent of their contribution, if any, remains to be determined.

#### Plasma dilution.

During pregnancy, blood plasma volume increases by nearly 50%, which could lead to dilution of blood proteins (34). However, in rodent studies, the decrease in maternal serum hepcidin was equivalent to the decrease in maternal liver *Hamp* mRNA (112), indicating that plasma dilution alone does not account for decreased hepcidin levels observed during pregnancy. It remains to be determined whether abnormally elevated hepcidin levels during pregnancy could be indicative of a failure of plasma dilution.

#### GDF15.

At relatively high concentrations, growth differentiation factor 15 (GDF15) [also known as macrophage inhibitory cytokine-1 (MIC-1) or placental transforming growth factor beta (PT-GFB)] has been shown to suppress hepcidin in vitro in primary human hepatocytes (129). GDF15 is highly expressed by the placenta (77). In humans, maternal serum concentrations of GDF15 increase with pregnancy progression, with an approximate 10-fold increase in the first trimester compared with nonpregnant controls (36). However, the same study reported no correlation between hepcidin and GDF15. The hepcidin response to increasing GDF15 concentrations appears to be biphasic, with hepcidin suppression seen only at very high concentrations of GDF15 (129), making it unlikely that GDF15 mediates the suppression of hepcidin in pregnancy.

#### Soluble hemojuvelin.

Soluble hemojuvelin (sHJV) is generated by proteolytic cleavage of the glycosylphosphatidylinositol-linked membrane form of hemojuvelin. sHJV suppresses hepcidin by antagonizing BMP-SMAD signaling, presumably by binding and sequestering bone morphologic proteins (5). Interestingly, during human pregnancy, maternal sHJV concentrations increase significantly in the third trimester and correlate with hepcidin (36). Authors of the study postulate that tissue hypoxia induced by fetal growth could upregulate HJV cleavage, resulting in the increase in the soluble form; however, systematic studies are needed to determine if sHJV regulates hepcidin during pregnancy.

### Fetal Hepcidin Regulation

The fetal liver can produce hepcidin during embryonic development and could thus regulate placental iron transport and fetal iron homeostasis. Indeed, an increase in fetal hepcidin in mice, through transgenic *Hamp* overexpression or as a result of matriptase-2 (*Tmprss6*) mutations (32, 100, 141), caused dose-dependent fetal iron-deficiency anemia and even resulted in fetal death. Thus, regulation of fetal hepcidin during pregnancy could be important for maintenance of fetal iron homeostasis.

Measurement of fetal hepcidin in mice in a healthy pregnancy indicated that fetal hepcidin expression is very low (100, 111). However, fetal hepcidin is responsive to stimuli (Figure 1) and can be suppressed by iron deficiency (115) or induced by iron loading (71). Fetal hepcidin is also regulated by ERFE during iron-deficiency anemia, and, in a mouse study, lack of fetal ERFE resulted in increased hepcidin, decreased fetal iron endowment, and diminished fetal erythropoiesis and caused fetal tissue iron deficiency (115).

Fetal hepcidin can also be potently induced by inflammation (39). Endotoxin-induced intra-amniotic inflammation in the rhesus macaque model of pregnancy greatly increased fetal plasma hepcidin and caused hypoferremia in the fetus, without affecting maternal plasma hepcidin (39). Intra-amniotic infection had a similar effect in human preterm neonates, causing increased cord blood hepcidin concentrations and hypoferremia (39).

Of note, mouse studies showed that fetal hepcidin is strongly increased by labor (100), highlighting the need for caution when interpreting cord blood measurements in human studies.

## PLACENTAL IRON TRANSPORT

Iron is transported unidirectionally from the maternal to fetal circulation across the placental syncytiotrophoblast, the layer that mediates nutrient and waste exchange (reviewed in 114). Over an average singleton human pregnancy, the placenta transports approximately 270 mg of iron to the fetus and retains approximately 45 mg (8, 13, 37, 139).

### Placental Import of Transferrin-Bound Iron

**Figure 2:**
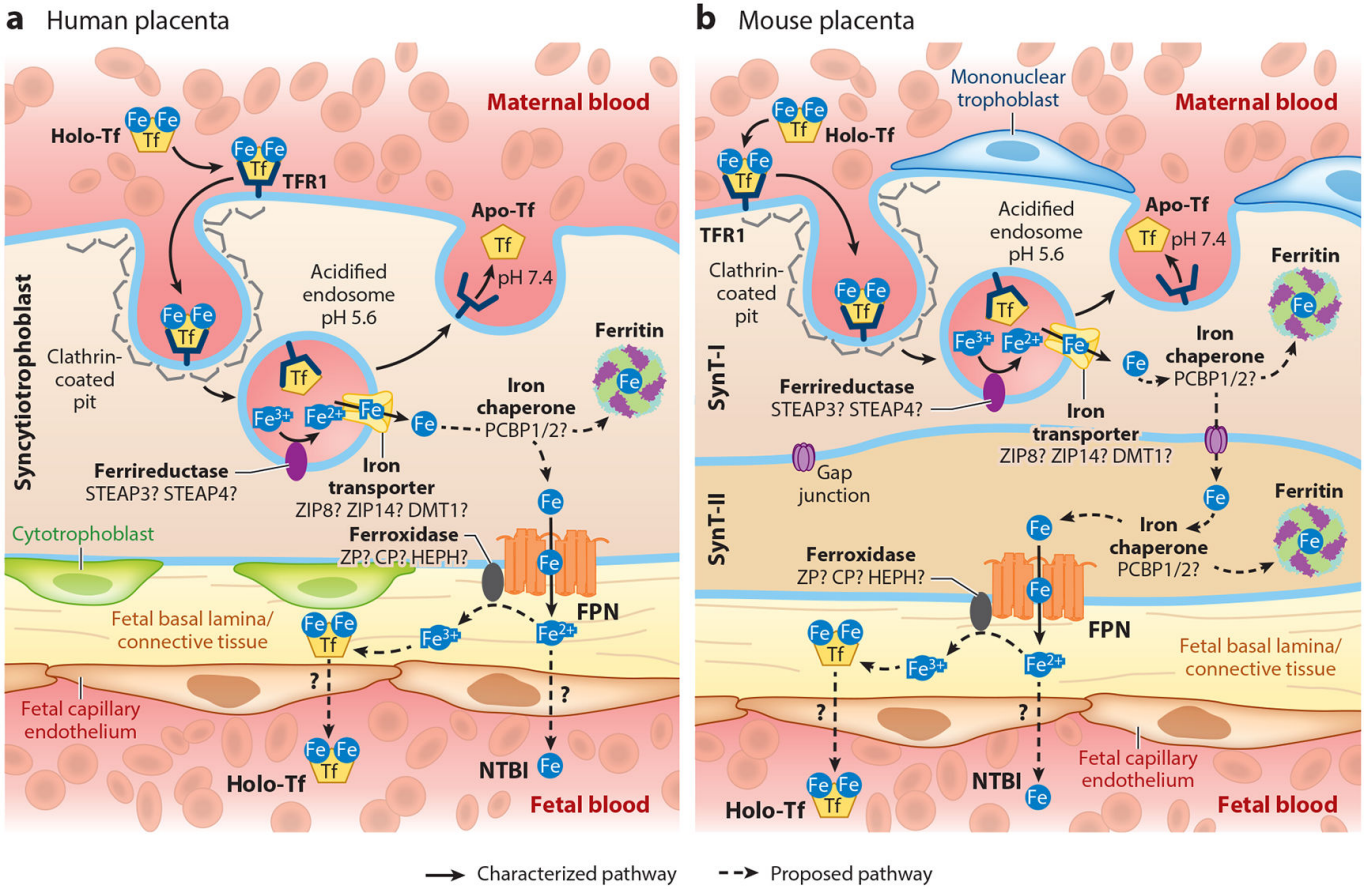
Iron trafficking across the syncytiotrophoblast. (*a*) Human placenta, with a single layer of syncytiotrophoblast. (*b*) Mouse placenta, with two syncytiotrophoblast layers (SynT-I and SynT-II). In both humans and mice, transferrin-bound iron (holo-Tf) from the maternal circulation binds to transferrin receptor TFR1, expressed on the apical membrane of the placental syncytiotrophoblast (SynT-I in mice). The iron-transferrin-receptor complex is internalized via clathrin-mediated endocytosis, and ferric iron (Fe^3+^) is released from transferrin (Tf) in acidified endosomes. The apo-Tf/TFR1 complex is recycled back to the cell surface. Fe^3+^ in the endosome is thought to be reduced to ferrous iron (Fe^2+^) by a ferrireductase and exported into the cytoplasm through an endosomal iron transporter. Cytoplasmic Fe may be chaperoned, possibly by PCBP1 or PCBP2, either to ferritin for storage or to ferroportin (FPN) on the basal membrane (SynT-II in mice) for export toward the fetal circulation. In the mouse placenta (*b*), it is unknown how Fe is transported from SynT-I to SynT-II, but it likely occurs through gap junctions. The fate of iron following export through ferroportin is unclear; it may enter the fetal circulation as nontransferrin bound iron (NTBI) or be oxidized to Fe^3+^ by ferroxidases and loaded onto transferrin prior to reaching the fetal circulation. Figure adapted with permission from Reference 114.

Uptake of transferrin (Tf)-bound iron from the maternal circulation is mediated by the iron importer transferrin receptor 1 (TFR1) on the apical membrane of the placental syncytiotrophoblast (9, 18, 49), facing the maternal circulation (Figure 2). Fe^3+^-transferrin binds to TFR1, and the complex is internalized by clathrin-mediated endocytosis. In the endosome, the acidic environment (pH 5.4) facilitates dissociation of Fe^3+^ from transferrin. Fe^3+^ is reduced to Fe^2+^ by ferrireductases, possibly by STEAP3 (six-transmembrane epithelial of prostate) or STEAP4, which are expressed in mouse and human placentae (101). Following the release of iron, the apotransferrin-TFR1 complex is recycled back to the membrane. The pH of the extracellular space (pH 7.4) facilitates dissociation of apotransferrin from TFR1, releasing apotransferrin back into the circulation. TFR1 is then again available for uptake of iron-rich holotransferrin.

### Transplacental Iron Trafficking

It is not fully understood how Fe^2+^ is subsequently transported across the endosomal membrane into cytoplasm. In erythroid cells, DMT1 (SLC11A2) transports iron across the vesicular membrane (56). In humans, DMT1 localizes to both the apical and basal membranes of the syncytiotrophoblast (49, 56, 85). In the mouse placenta, DMT1 partially colocalizes with endocytosed Tf-TFR1, suggesting a role in iron export from the endosome (19). However, DMT1 is dispensable in the placenta, as genetic ablation in mice does not affect iron endowment (56). Other potential transporters include ZIP8 (Zrt/Irt-like protein) and ZIP14, members of the SLC39A (solute carrier family 39A) family. ZIP8 (SLC39A8) and ZIP14 (SLC39A14) are abundantly expressed in placenta (136). Similar to DMT1, ZIP14 is dispensable in the placenta, as null mice have normal iron stores (64). In contrast, ZIP8 deficiency causes anemia and lethality of mouse embryos (43), showing that ZIP8 is essential for development. The contribution of ZIP8 to placental iron metabolism and transport remains to be elucidated.

Within the syncytiotrophoblast, iron is stored as ferritin or is exported across the basal syncytiotrophoblast membrane to the fetal circulation. In most tissues, ferritin iron can be released through the process of ferritinophagy (116), and this may also occur in the placenta, providing additional iron for export to fetal blood. How iron trafficking occurs within the syncytiotrophoblast is unknown but may involve iron chaperones PCBP1 and PCBP2 (poly(rC)-binding proteins), like in other cell types (108, 144).

### Placental Iron Export

Iron is transported out of the syncytiotrophoblast by the sole iron exporter ferroportin (9). Ferroportin is expressed on the basal membrane of human and mouse placental syncytiotrophoblasts facing the fetal circulation (112) (Figure 2) and is essential for placental iron transport and fetal iron endowment. Global ablation of the ferroportin gene *Slc40a1* is lethal to mouse embryos by E9.5 (31), and *Slc40a1* hypomorphs are severely anemic (92, 97). Selective expression of *Slc40a1* in the placenta but not the embryo rescued lethality, demonstrating the placenta-specific role of ferroportin in iron transport (31). How iron reaches the fetal circulation after exiting the syncytiotrophoblast and how it crosses the fetal endothelium is unclear. Iron undergoes oxidation to the ferric form before loading onto fetal transferrin. Ceruloplasmin (CP), hephaestin (HEPH), and zyklopen (ZP) are multicopper ferroxidases expressed in the placenta (24, 55). Knockout mouse models indicate their individually dispensable roles in the placenta (42, 59, 63, 135). Their specific functions in placental iron transport remain to be clarified.

### Differences Between Human and Mouse Placentae

Animal models are invaluable for understanding iron transport mechanisms, especially during pregnancy. The availability of a wide array of genetically modified mice makes them a commonly utilized animal model. Specifically for pregnancy studies, key iron regulatory and transport mechanisms are similar between human and mouse placentae (28). However, there are important anatomical, structural, and cellular differences, including differences in the number of fetuses, uterine shape, and the site of progesterone synthesis in late gestation (104). Structurally, both human and mouse placentae are hemochorial (i.e., maternal blood is in direct contact with chorionic villi); however, the syncytiotrophoblast, the cells responsible for nutrient and waste transfer between mother and fetus, differs. The human syncytiotrophoblast is a single cell layer, whereas in the mouse placenta it is composed of two distinct layers (SynT-I and SynT-II) (107) that communicate via gap junctions (Figure 2). Although the key placental iron transporters, transferrin receptor and ferroportin, are mechanistically required for both human and mouse placental iron uptake and export, noted differences between the species and possible redundancies in iron transport proteins have made it challenging to analyze iron handling within the placenta.

### Transport of Alternative Iron Species Across the Placenta

Holotransferrin from the maternal circulation is thought to be the main iron species taken up by the placenta (Figure 2). In mice, global *Tfrc* deficiency resulted in embryonic anemia and lethality before E12.5 (81), but it is not clear whether this was a result of impaired placental transport or impaired fetal erythroid iron uptake or both. All *Tfrc*-deficient embryos were anemic (81), but evidence of RBC production was observed in some *Tfrc*-deficient embryos, suggesting that iron sources other than transferrin-bound iron may temporarily support erythropoiesis, albeit ineffectively. Iron may also be complexed to other plasma molecules such as citrate, other organic anionic acids, and albumin, forms collectively referred to as nontransferrin bound iron (NTBI), usually when transferrin is highly saturated. It is unlikely that maternal circulation in healthy pregnancy contains NTBI considering the relatively low transferrin saturation. However, it is possible that iron exported out of the syncytiotrophoblast to the fetus can exist in the NTBI form (33) (Figure 2) and that it can support embryogenesis to a certain extent. Furthermore, it is unknown whether heme or ferritin crosses the placenta. Proteins involved in heme transport and metabolism that are expressed in the placenta include heme exporters FLVCR1 and FLVCR2 (feline leukemia virus subgroup C receptor-related proteins 1 and 2) (73), heme-hemopexin receptor LRP1 (LDL receptor-related protein 1) (20), and heme oxygenases (89). Animal studies demonstrated the transport of labeled ferritin to the fetus (75), although the physiological relevance of this is unclear, as circulating ferritin is typically iron poor. Ferritin uptake by the placenta might involve TFR1 (83) and/or SCARA5 (scavenger receptor class A member 5) (82). Although the transporters for NTBI, ferritin, and heme are expressed in the placenta, it is unknown whether any of these molecules represent a significant iron source for placental transport or fetal development.

## PHYSIOLOGICAL AND PATHOLOGICAL REGULATION OF PLACENTAL AND FETAL IRON HOMEOSTASIS

Iron delivery to the fetus is dependent on transport across the placenta. The human fetus obtains 80% of its iron endowment in the third trimester of pregnancy (13, 37, 139); thus, placental iron transport during this period is expected to be maximal. Indeed, studies in human and mouse placentae demonstrate increased expression of iron transporters as pregnancy progresses (15, 112), presumably to facilitate iron delivery to the fetus when growth is maximal. Normal body iron content in healthy term newborns is 75 mg/kg, with 1.35 mg of iron/kg/day accruing in the third trimester (139). Most fetal iron is in oxygen-transporting hemoglobin (75–80%), and the rest performs many diverse functions in iron-containing proteins in tissues (10%) or is stored as ferritin (10–15%). Cord blood ferritin and hepcidin concentrations were reported to increase with gestational age (87, 90, 123), in agreement with maximal fetal iron accrual in late pregnancy; yet, one study reported no correlation of cord blood hepcidin or ferritin with gestational age in adolescent pregnancies (78). The relationship between body iron, hepcidin, and ferritin levels is well established (46); however, interpretation of these measurements in pregnancy may be confounded by the physiologic effects of labor and delivery or by the presence of inflammation in complicated pregnancies (39, 78, 79, 87, 127). Although not all of the mechanisms are well understood, it has become clear that placental and fetal iron homeostasis is regulated by maternal, placental, and fetal signals that alter iron availability and distribution, but the relative contribution of these signals varies in different pathophysiological conditions.

### Inflammation and Hepcidin

Placental iron transport is reliant on iron availability in the maternal circulation, which is ensured by the suppression of the maternal hormone hepcidin (112). Placental iron transfer to the fetus is inversely corelated with maternal hepcidin concentrations (148), and conditions such as inflammation, which induce maternal hepcidin, would be expected to limit iron availability for placental iron transfer. Indeed, in mice, induction of acute systemic inflammation by lipopolysaccharide (LPS) injection overcomes the pregnancy-dependent suppression of maternal hepcidin, causing hypoferremia in the dam and embryo (39, 112). Independently of inflammation, prolonged elevations in plasma hepcidin elicited by administering a hepcidin mimetic throughout pregnancy in mice caused severe iron restriction and anemia in dams and embryos (111), confirming that elevated maternal hepcidin itself is sufficient to cause adverse pregnancy outcomes. In healthy human pregnancies, hepcidin did not correlate with inflammatory markers (121) except in some deliveries (79), suggesting that mild inflammation during pregnancy does not appreciably affect hepcidin production. However, chronic maternal inflammation caused by obesity is associated with increased risk of infant anemia (146), possibly through prolonged elevations in hepcidin levels in obese women causing iron restriction (23, 29, 133). Other inflammatory conditions such as preeclampsia are also associated with increased hepcidin levels (131), potentially affecting iron availability for placental transfer. Importantly, in a cohort of pregnancies complicated by inflammation and nutrient deficiencies, higher maternal hepcidin was identified as a main determinant of intrauterine growth restriction (53), suggesting that hepcidin-mediated iron restriction, particularly when chronic, may be an important pathological factor in adverse pregnancy outcomes.

Given the orientation of placental ferroportin on the basal membrane facing the fetal circulation, placental ferroportin is exposed to regulation by fetal hepcidin. Indeed, transgenic embryos overexpressing hepcidin are iron deficient and anemic compared with their wild-type littermates (100). Furthermore, embryos with null mutations in *Tmprss6,* a repressor of hepcidin expression, have 60-fold increased hepcidin levels compared with those of control embryos, lower iron stores, and lower expression of placental ferroportin, showing that fetal hepcidin can regulate placental ferroportin (141). However, in the absence of inflammation, placental and fetal hepcidin concentrations under physiologic conditions are too low to regulate iron transfer across the placenta (71, 100, 112, 141). Nevertheless, certain pathological conditions such as intra-amniotic infection or inflammation can induce fetal hepcidin sufficiently to alter fetal iron distribution (127) (Figure 1d). In macaque pregnancies complicated by intra-amniotic inflammation where inflammation was restricted to the fetal compartment, maternal hepcidin was not elevated, whereas fetal hepcidin was increased and associated with fetal hypoferremia (39), demonstrating that in pathologically inflamed pregnancies the fetus can regulate its own iron homeostasis by inducing hepcidin. Whether this elevated fetal hepcidin regulates both placental iron transport and systemic fetal iron flows remains to be confirmed. In human pregnancies with intra-amniotic infection or inflammation, a similar fetal response was observed where human fetuses exposed antenatally to intra-amniotic infection had elevated cord blood plasma hepcidin levels and hypoferremia. In general, cytokine-driven increases in hepcidin and consequent hypoferremia (98) are important host defense mechanisms to prevent the spread of pathogenic bacteria by limiting iron availability and, in particular, decreasing the concentration of NTBI, a form highly accessible to many pathogens (125, 126). Thus, the ability of the fetus to respond to inflammatory signals by sequestering iron away from extracellular bacteria may be an important protective mechanism during intra-amniotic infections, although chronic elevation of fetal hepcidin would likely be detrimental by causing fetal iron restriction and anemia.

### Iron Deficiency

Pregnancy is a major challenge to systemic iron homeostasis, and women with insufficient iron stores before pregnancy are at increased risk of developing iron deficiency and anemia. Maternal iron deficiency during pregnancy compromises fetal and neonatal iron endowment and is associated with fetal iron deficiency and anemia in humans and animal models (1, 26, 39, 44, 52, 112), as well as increased maternal morbidity and mortality, preterm birth, low birth weight, cognitive defects in newborns, and impaired immune function (4, 25, 27, 48, 93, 117, 120). During iron-deficiency anemia, maternal hepcidin is further suppressed to allow iron absorption and mobilization from stores, but this may not correct the problem if dietary iron availability and iron stores are already very limited. Similar to maternal hepcidin, fetal hepcidin also decreases in response to iron-deficiency anemia (26, 44) (Figure 1c), presumably to promote iron transfer across the placenta and mobilize iron from fetal stores. Fetal hepcidin suppression during iron deficiency anemia is at least in part mediated by fetal ERFE (111, 115).

In iron-deficient pregnancies, the placenta itself undergoes adaptations, and increases in placental TFR1 were observed in both humans and animal models (12, 26, 44, 112, 147). Increased placental TFR1 in iron-deficient pregnancies reflects placental sensing of limited iron availability, a response mediated by IRP1 and IRP2 (iron regulatory proteins 1 and 2), which regulate proteins involved in iron uptake, storage, and export (140). Maternal obesity is also associated with increases in placental TFR1, likely because low iron stores are more common in obese women rather than as a direct effect of obesity on placental TFR1 (47). With severe maternal iron-deficiency anemia in mice, and also in isolated primary human trophoblasts in vitro when exposed to iron chelator, the trophoblast decreases the iron exporter ferroportin, an adaptation mediated by IRP1 (112), which would lead to a counterintuitive iron retention in the placenta (112). Indeed, in the mouse model with severe maternal and fetal iron deficiency, placental iron content was relatively stable throughout gestation except for only a small decrease in placental iron at E18.5. Similarly in rats, placentae from iron-deficient pregnancies had lower nonheme iron content but similar total and heme iron content compared with that of iron-adequate pregnancies (26). Maintaining placental iron content during limited iron availability at the expense of fetal iron deficiency appears counterintuitive but may be important for preserving a broad array of placental metabolic functions (112), which would indirectly benefit the fetus. Indeed, causing iron deficiency in isolated human trophoblast in vitro greatly impaired mitochondrial respiration, which would be expected to be detrimental to a tissue that is as highly metabolically active as placenta (112), highlighting the importance of placental adaptation mechanisms that promote placental iron acquisition and retention to maintain the placenta’s own homeostasis.

### Iron Supplementation and Excess

Iron supplementation is generally recommended to prevent the adverse effects of iron deficiency and anemia. Maternal iron supplementation is shown to improve anemia and ferritin levels in pregnant women but had little effect on cord blood ferritin at delivery. However, infants of mothers who were iron supplemented during pregnancy had a detectable improvement in serum ferritin and anemia several months after delivery (96, 103).

In mice, embryos from iron-supplemented dams (from single injection of iron dextran or fed a 1% carbonyl iron diet) were mostly protected from iron overload due to the induction in maternal hepcidin (112), although placentae were iron loaded with dietary iron supplementation. In hepcidin-deficient dams, even embryos become iron loaded, and the degree of iron loading was dependent on maternal iron status (39), with increased maternal iron loading leading to greater embryo overload. Compared with those of embryos, amniotic fluid iron concentrations were unaffected by maternal iron status but increased with gestational age (39). The biological implications of these changes are unknown.

Pregnancy-dependent suppression of maternal hepcidin would promote the rapid absorption of commonly prescribed iron supplements, potentially exposing normal pregnancies to iron excess and NTBI. Indeed, NTBI is detectable in plasma shortly after ingesting iron supplements (17), but whether NTBI affects the placenta or fetal tissues is unknown. Maternal exposure to NTBI could also occur in pregnancies complicated by the iron-overload disorder hereditary hemochromatosis or the blood disorder β-thalassemia, where excess iron absorption and the appearance of NTBI in circulation can lead to organ damage including diminished fertility and increased pregnancy complications. In β-thalassemia, transfusions and cessation of chelation during pregnancy would further increase the risk of generating NTBI in maternal circulation (21, 128). Women of reproductive age and pregnant women with milder forms of hereditary hemochromatosis are generally protected from iron overload because of menstruation or pregnancy losses, and pregnancy outcomes are favorable when properly managed (6). Pregnant women with β-thalassemia, according to several studies, have increased risk of adverse pregnancy outcomes, including intrauterine growth restriction and low birth weight, prematurity, abortion, and intrauterine fetal death (41, 132). High iron levels can cause tissue damage by the generation of reactive oxygen species (143). However, to what extent the adverse outcomes are related to maternal anemia versus iron excess in the maternal, placental, or fetal compartment remains to be determined.

Although maternal iron supplementation is effective in treating iron deficiency and anemia, there is a U-shaped curve relating maternal iron status or iron supplementation to the frequency of adverse consequences in infants and young children. Maternal and neonatal iron deficiency is associated with impaired cognitive, motor, and behavioral development of children (88). On the other hand, elevated maternal serum ferritin has also been associated with preterm birth, low birth weight, gestational diabetes, and neurodevelopmental deficiencies in children (14, 51, 57, 74, 76, 119). However, the attribution of increased serum ferritin to iron overload requires great caution because increased ferritin can also result from inflammation. Excess iron supplementation in infants was associated with decreased growth, along with impaired cognitive and motor function (86).

In mouse models, maternal iron excess in the absence of inflammation did not cause adverse pregnancy outcomes such as fetal lethality or gross morphological malformations, although a more detailed analysis of the outcomes in offspring development is needed (38). Importantly, it was the combination of maternal systemic inflammation and excess iron that had dire consequences for fetal development. Iron excess (as a result of hepcidin deficiency or feeding with a 0.25–0.5% carbonyl iron diet) in dams with systemic inflammation caused embryo malformations and embryo demise (38). This was observed with both the model of acute systemic inflammation (subcutaneous LPS injection) and the model of obesity, suggesting that excessive iron supplementation could be harmful in pregnant women with underlying inflammation. Whether iron deficiency similarly potentiates inflammation-induced fetal injury remains to be determined. Overall, these studies underscore the importance of accurately identifying and treating iron disorders during pregnancy for optimal health in infants and young children.

## CONCLUSIONS AND FUTURE DIRECTIONS

Iron is essential for a healthy pregnancy to support the development of the placenta, fetal growth, and maternal physiologic adaptations, including expansion of maternal RBC mass. Like in the nonpregnant state, regulation of maternal iron homeostasis is controlled by hepcidin, and maternal hepcidin suppression during pregnancy is essential for adequate iron supply to the fetus. Abnormal maternal iron status at either extreme, either deficient or excess iron, has negative consequences for both the mother and the baby. Although recent advancements have contributed substantially to our understanding of maternal, placental, and fetal iron homeostasis during pregnancy, important gaps remain. Further research is needed to fully characterize iron regulation during pregnancy including identifying the iron-independent mechanism of hepcidin suppression in pregnancy, elucidating the molecular mechanisms of placental iron transport, defining the iron species contributing to fetal development, and defining the cellular consequences of iron deficiency and iron excess in the mother, the placenta, and the fetus and their association with adverse outcomes.

## Figures and Tables

**Table 1:** Physiological iron balance in menstruating and pregnant females by gestation

Iron fate	Menstruating females (28 days)	First trimester (weeks 0–13; 13 weeks total)	Second trimester (weeks 14–26; 13 weeks total)	Third trimester (weeks 27–40; 14 weeks total)	Pregnancy sum (weeks 0–40; 40 weeks total)
Basal iron losses	23 mg (0.8 mg/day)^a^	73 mg (0.8 mg/day)^a^	73 mg (0.8 mg/day)^a^	78 mg (0.8 mg/day)^a^	224 mg
Menstruation	13 mg^a^	NA	NA	NA	NA
Placental iron	NA	NA	16 mg (0.2 mg/day)^d,e^	30 mg (0.3 mg/day)^b,d,f^	46 mg
Fetal iron	NA	NA	60 mg (0.7 mg/day)^b,c^	210 mg (2.1 mg/day)^b,c^	270 mg
Expansion of maternal RBC mass	NA	NA	112 mg (1.2 mg/day)^g,h^	338 mg (3.5 mg/day)^g,h^	450 mg^h^
Total iron needs	36 mg (1.3 mg/day)	73 mg (0.8 mg/day)	261 mg (2.9 mg/day)	656 (6.7 mg/day)	990 mg
Delivery blood loss	NA	NA	NA	NA	150 mg^g^
RBC mass contraction after delivery	NA	NA	NA	NA	−450 mg^g^
Net iron loss	NA	NA	NA	NA	690 mg

On the basis of data reported by ^a^Hallberg & Rossander-Hulten (58), ^b^Widdowson & Spray (139), ^c^Alexander et al. (2), ^d^Barad et al. (8), ^e^Hecht et al. (61), ^f^Hayward et al. (60), ^g^Bothwell (13), and ^h^Hytten (69), assuming a 55-kg woman.

Menstruation calculation (58): Hb, 135 g/L blood; Fe,3.34 mg/g Hb.

Fetal iron: iron content/g fetus weight (139); average fetal weights in second trimester (2, 110) and third trimester (2).

Placental iron: iron content/g placenta weight (71.1 μg/g) (8); average placenta weight in second trimester (61) and third trimester (8, 60, 139).

Maternal RBC expansion: total iron required (13); second and third trimesters area under curve calculation assuming a linear increase in requirement (69). Abbreviations: Hb, hemoglobin; NA, not applicable; RBC, red blood cell.
